# Psychosocial school factors and mental health of first grade secondary school students—Results of the Health Behaviour in School-aged Children Survey in Serbia

**DOI:** 10.1371/journal.pone.0293179

**Published:** 2023-11-09

**Authors:** Dragana Skoric, Jelena Gudelj Rakic, Verica Jovanovic, Dusan Backovic, Ivan Soldatovic, Jelena Ilic Zivojinovic

**Affiliations:** 1 Centre for Hygiene and Human Ecology, Institute of Public Health of Serbia "Dr Milan Jovanovic Batut", Belgrade, Serbia; 2 Centre for Health Promotion, Institute of Public Health of Serbia "Dr Milan Jovanovic Batut", Belgrade, Serbia; 3 Centre for Disease Control, Institute of Public Health of Serbia "Dr Milan Jovanovic Batut", Belgrade, Serbia; 4 Institute of Hygiene and Medical Ecology, Faculty of Medicine, University of Belgrade, Belgrade, Serbia; 5 Institute of Medical Statistics and Informatics, Faculty of Medicine, University of Belgrade, Belgrade, Serbia; Chiba Daigaku, JAPAN

## Abstract

This cross-sectional study aimed to investigate the association between psychosocial school factors and life satisfaction, symptoms of depression and psychosomatic health complaints among first grade secondary school students in Serbia. We analysed data from the 2018 Health Behaviour in School-aged Children (HBSC) study in the Republic of Serbia. Analyzed psychosocial school factors included satisfaction with school, schoolwork pressure, teacher support, classmate support and being bullied at school. Life satisfaction was assessed by the 11-step Cantril’s ladder (cutoff >5). Symptoms of depression were measured by the Center for Epidemiologic Studies Depression Scale (CESD-10) and psychosomatic health complaints by using the HBSC symptom checklist. Univariable and multivariable binary logistic regression was used to determine independent predictors of students’ life satisfaction, symptoms of depression and psychosomatic health complaints in the school environment, while also considering their socio-demographic characteristics and perceived family and friend support. The study included 1605 students (average age 15.26 ±0.44 years), of whom 50.3% were females. Results from the binary logistic regression analyses showed that life satisfaction was positively related to school satisfaction and classmate support, and negatively to being bullied at school. Symptoms of depression were positively associated with schoolwork pressure and being bullied at school, and negatively with teacher and classmate support. All analyzed factors of the school environment were significantly related to psychosomatic health complaints, whereby schoolwork pressure and being bullied at school were positively associated, while teacher and classmate support and satisfaction with school were negatively associated. Given the established association of psychosocial school factors with mental health, there is a need for targeted measures both at school and community level with the aim of improving social support in the school environment, reducing schoolwork burden and preventing bullying at school, potentially resulting in the overall improvement of mental health of the first grade secondary school students.

## Introduction

Schools represent an environment in which students spend a significant part of their time during childhood and adolescence. A supportive school environment can contribute to the development of healthy lifestyles and good mental health of students [1].

The definition of mental health has changed over time from negative aspects, through positive, to an integrative concept, where it is described as a continuum that moves from mental problems to well-being, or is viewed from multiple dimensions [2]. Namely, the same person can simultaneously have a high level of well-being, but also mental problems, so it is necessary to look at mental health in the widest possible context. The World Health Organisation (WHO) also emphasizes that people with a mental health disorder are more likely to have a low level of mental well-being, but this is not and may not always be the case [3]. In this sense, Ringdal et al. state the importance of examining the effects of potential predictors in identical models for both positive and negative aspects of mental health [2].

Life satisfaction represents an important aspect of subjective well-being, one of the most important components in modern models of mental health [4]. It is defined as “a general assessment that people make when considering their life as a whole” [5]. It is believed that the level of satisfaction with life can affect future health and even mortality [6]. There are numerous studies dealing with the determinants of life satisfaction, including those at the individual and family, school, community and national level. In the school environment, the literature data include the importance of satisfaction with school [4, 7], perceived schoolwork pressure, teacher [6, 8] and classmate support [6, 8], as well as being bullied at school [9–11].

Depression is one of the most common mental health disorders in adolescence with an estimated prevalence of 4–5% in middle and late adolescence [12]. In the school environment, factors related to depressive symptoms or to occurrence of depression in adolescence include stress related to school and academic success [2, 13], school connectedness [14], relationship with teachers [13, 14] and bullying [2, 12, 15, 16].

Estimated prevalence of psychosomatic health complaints in children and adolescents ranges between 10% and 25% [17]. Clinical manifestations in adolescents may be different when compared to adults. The most common symptoms in children are abdominal pain, headache, chest pain, fatigue, back pain, and breathing difficulties [17]. Symptoms vary and usually two or more occur at the same time and persist for longer periods of time. Previous research identified perceived schoolwork pressure, school satisfaction (including teacher and peer support) and bullying as factors in the school environment contributing to occurrence of psychosomatic health complaints [7, 10, 18–23].

Results from former research studies vary regarding the importance and influence of different factors in the school environment on mental health. For instance, some studies report that classmate support is significantly associated with psychosomatic health complaints [19, 21], while some did not find this association significant [22]. There are also different findings regarding the significance of classmate support in relation to symptoms of depression [24, 25]. Additionally, previous studies often do not include both positive and negative aspects of mental health. Literature review revealed only few studies that examined relationship between broader number of psychosocial factors, including bullying at school, with occurrence of psychosomatic health complaints [26], depression [2], and life satisfaction. Moreover, the effect of protective factors such as family or friend support was not included.

In the national context, there is a lack of epidemiological data on adolescents’ mental health and wellbeing and currently there are no national prevalence estimates of adolescents’ mental health problems. Furthermore, studies regarding mental health on a representative sample of this population are missing. A recent study indicates that less than 31% of Serbian youth aged 15–29 has within-the-normal-range stress level, 69% has anxiety and 52% has symptoms of moderate or severe depression [27]. Moreover, there is a lack of data on factors associated with their mental health and wellbeing. In Serbia according to our knowledge there are no studies on association between psychosocial factors in the school environment and mental health. Therefore, the aim of the current study was to examine the association of psychosocial factors in the school environment with life satisfaction, psychosomatic health complaints and symptoms of depression in the first grade secondary school students in Serbia. We hypothesized that there is an association between psychosocial factors of the school environment with these mental health parameters.

## Materials and methods

This cross-sectional study presents a secondary analysis of data obtained from the Health Behavior in School-aged Children (HBSC) Study in Serbia in 2018. The national survey was carried out using the International protocol of the HBSC study, in line with the WHO methodology described in more detail elsewhere [28, 29]. According to the protocol, study participants were 11, 13 and 15 year old students. The participants were recruited from the selected sample of schools who accepted to participate in the survey. Sampling frame was list of primary and secondary schools in Serbia. A stratified, multistage sampling approach was used for the selection of the survey sample. Independent samples were selected for each of the age groups. Estimated average number of students per class was 20. Sample included 64 school for each grade, i.e. age group—39 schools per each age group was selected as main sample and additional 25 replacement schools due to possibility that some students could refuse to fill in the questionnaire or that parents (or guardians) would not give their consent for children’s participation in the survey. After the initial school selection, list of classes for the defined age groups was made and using table of random numbers two classes for each age group were selected.

After sampling, selected schools were contacted first by mail informing them about the survey and asking their consent to participate in the survey. Replacements schools were included in case that school refused to participate, however replacement schools were selected so that they would resemble schools in original sample (according to size—number of students, region, etc.). In recruitment stage, after initial mail, schools were contacted by phone. Recruitment was done during March 2018. Data collection was done between April 16th and June 7th 2018 in selected schools during one school class.

The Ethical review board of the Institute of Public Health of Serbia consented to the implementation of the HBSC study in Serbia. A self-administered questionnaire was used with the parents’ (guardians’) consent that was collected by school Students completed questionnaire during one school class. Participation in the study was voluntary for the students, meaning that even though parents consented for their child to participate in the study, the adolescents could refuse to participate at the day of the survey. All students were free to end their participation in the survey at any time.

Participation in survey was anonymous and no personal information was collected. The completed questionnaires were put in closed envelopes. Authors had no access to information that could identify individual participants during or after data collection. The Ethical review board of the Institute of Public Health of Serbia also consented to perform this particular study (decision no. 3839/1 of June 22, 2021).

### Study population

In the HBSC study in Serbia in 2018, students enrolled in the fifth and seventh grade of the primary school and students in the first grade of secondary schools (according to school system in the Republic of Serbia) in selected schools and selected classes were provided information about the survey including anonymity and voluntary participation. Students with parental (or guardian) consent of selected classes who accepted to participate in the survey were eligible survey participants. There were 192 initially selected and 101 participating schools. Out of 384 classes, 200 participated. Out of 7680 students, 4028 participated. Refusals were either due to not having parental (or guardian) consent or due to absence because of illness on the day of survey.

In this study, we performed a secondary data analysis of the HBSC study in Serbia and target population in this particular study included only first grade secondary school students, since we were especially interested in the importance of psychosocial factors after the transition from primary to secondary school. Being a student of the first grade in secondary school was the only inclusion criterion and there were no exclusion criteria.

### Survey instrument

Survey instrument was standardized, self-administered questionnaire that included mandatory and optional questions. Students filled in the questionnaire during one school class. The questionnaire was anonymous.

The questions used for this analysis included demographic and socio-economic characteristics of the respondents, self-assessed health status, questions related to school, family, friends and peer violence/abuse.

### Measures

School type was differentiated based on whether the student attended grammar school or secondary vocational school.

Four statistical regions were identified: Belgrade, Vojvodina, Šumadija and Western Serbia, Southern and Eastern Serbia.

Socio-economic status was determined based on the Family Affluence Scale (FAS III), a composite indicator which is used to measure objective socio-economic status within the HBSC study [30]. It is determined based on the following six questions: “How many computers does your family own” (none– 0; one– 1; two– 2; more than two—3), “Does your family own a car, a van or a truck” (no– 0; yes, one– 1; yes, two or more—2), “Do you have your own bedroom for yourself” (no-1; yes-2), “Does your family have a dishwasher at home” (no– 0; yes -1), “How many bathrooms (room with a bath/shower or both) are in your home” (none– 0; one– 1; two– 2; more than two -3), “How many times did you and your family travel for vacation/holiday outside of Serbia in the last 12 months” (not at all– 0; once– 1; twice– 2; more than twice—3). By summing up these points, the score of the perceived family’s socio-economic status was calculated (ranging from 0 to 13) and this variable was further analyzed as numerical.

School satisfaction was estimated based on the question: “How do you like school at present” with answer options ranging from 1 –“I like it a lot” (highest level of school satisfaction) to 4 - “I don’t like it at all” (lowest level of school satisfaction). This variable was dichotomized so that the answers “I like it very much” and “I like it a bit” denote school satisfaction and the answers “I don’t like it very much” and “I don’t like it at all” denote school dissatisfaction.

Schoolwork pressure was determined by the question “How pressured do you feel by the schoolwork you have to do?” Answer options range from 1 –Not at all (lowest level of schoolwork pressure) to 4-A lot (highest level of schoolwork pressure). Answers were grouped so that a student was considered to be pressured by schoolwork if the answer was “Yes, a lot” and “Yes, very much”, as opposed to “Yes, a little” and “Not at all”.

Classmate support was determined based on answers from a 5-point Likert scale of agreement (from “strongly disagree” to “strongly agree”) with the following statements: “Students in my class like to be together”, “Most students in my class are friendly and want to help”, “Other students accept me as I am”. A sum-score was generated from the responses to the above three items ranging from 3 to 15. The final score was classified as high or low classmate support based on a cut-off value of ≥2.5 [31].

Teacher support was determined based on answers from a 5-point Likert scale of agreement (from “strongly disagree” to “strongly agree”) with the following statements: “I feel that the teachers accept me as I am”, “I feel that the teachers (professors) take care of me as a person”, “I have great confidence in my teachers”. The same scoring system was applied as for classmate support [31].

Being bullied at school (bullying victimization) was estimated by the question “How often have you been bullied at school in the past couple of months” with the possible answers “I have not been bullied at school in the past couple of months”, “It has happened once or twice”, “2 or 3 times a month”, “About once a week” and “Several times a week”. Responses were grouped and dichotomized as “not bullied” (if answered I have not been bullied) and “bullied” (if answered all other answer options).

Life satisfaction was determined based on Cantrill’s scale: “Here is a picture of a ladder. The top of the ladder “10” is the best possible life for you and the bottom “0” is the worst possible life for you. In general, where on the ladder do you feel you stand at the moment?”, where respondents indicated their life satisfaction on a scale from zero to 19. Cut-off value of ≥6 was used for categorization of respondents with high and low life satisfaction [32].

The presence of depressive symptoms was evaluated by using Short depression scale of the *Center for Epidemiologic Studies Depression Scale (CESD-10)* [33], which consists of 10 statements about behaviours or feelings: “I was bothered by things that usually don’t bother me”, “I had trouble keeping my mind on what I was doing”, “I felt depressed”, “I felt that everything I did was an effort”, “I felt hopeful about the future”, “I felt fearful”, “My sleep was restless”, “I was happy”, “I felt lonely”, “I could not “get going”“. Available answer categories were as follows: “Rarely or none of the time (less than 1 day)”, “Some or a little of the time (1–2 days)”, “Occasionally or a moderate amount of time (3–4 days)”,“All of the time (5–7 days)”. The total score is calculated by summing up the 10 items. A score of 10 or higher out of 30 is the cut-off for clinically significant depressive symptoms. Based on this value, a dichotomization was made into subjects with and without symptoms of depression.

Psychosomatic health complaints were determined using the HBSC symptom checklist, which includes the frequency of the following complaints in the last six months: headache, stomachache, backache, feeling low, irritability or bad temper, feeling nervous, difficulties in getting to sleep, feeling dizzy. Possible answers were: “About every day”, “More than once a week”, “About every week”, “About every month”, “Rarely or never”. Students who had two or more symptoms (complaints) at the same time at least once a week were classified as having psychosomatic health complaints [18].

Perceived family and friend support was evaluated based on two subscales constructed of the Multidimensional Scale of Perceived Social Support (MSPSS). Items on family support measure the perceived availability of emotional support and help within the family and respondents stated agreement with the statements on a 7-point Likert scale, ranging from “very strongly disagree” to “very strongly agree”. The statements were as follows: “My family really tries to help me”, “I get the emotional help and support I need from my family”, “I can talk about my problems with my family”, “My family is willing to help me make decisions”. First the sum of responses was calculated, then its mean value. A cut-off score value of ≥5.5 points [31, 34] was considered as high support.

Perceived friend support was assessed the same as perceived family support by using a 7- point Likert response scale. The statements were: “My friends really try to help me”, “I can count on my friends when things go wrong”, “I have friends with whom I can share my joys and sorrows”, “I can talk about my problems with my friends”. A cut-off score of 5.5 points [34] was considered as high support.

Missing data for one or more statements for assessing classmate and teacher support, and friend and family support was considered as a missing value. Furthermore, missing data for one or more of the eight listed health complaints was considered as a missing value. Regarding the symptoms of depression, observations with two or more missing responses were not included in the analysis, while in the case of one missing response, that response was replaced by the mean value [35].

### Statistical analysis

Categorical variables were presented as numbers and percentages, while continuous variables were presented as means and standard deviations. Chi square test and the two tailed t test were used to test the difference in life satisfaction, the frequency of psychosomatic health complaints and depressive symptoms in relation to the observed factors of the school environment, demographic and socio-economic factors. Also, the chi-square test was used to test the difference in psychosocial factors in relation to the type of school. In addition, univariable and multivariable binary logistic regression was used to determine the association of psychosocial factors of the school environment with life satisfaction, depressive symptoms, and psychosomatic complaints. The outcome variables were: life satisfaction (high/low), psychosomatic health complaints (yes/no) and depressive symptoms (yes/no). Apart from psychosocial school factors, we also included demographic and socio-economic characteristics of the study participants, as well as perceived family and friend support into regression analyses due to their previously reported significance in the literature. Variance inflation factor—VIF value of 5 was used as a criterion for examining collinearity of independent variables. Independent variables that were significant at the p value of <0.1 in the univariable analysis were included in the models using the enter and backward methods. A p value of <0.05 was used as the minimum level of significance throughout the analysis. Statistical analysis was performed using the IBM SPSS Statistics, Version 20.0 (IBM Corp., Armonk, NY, USA).

## Results

In this study, 1605 students were included (average age 15.26 ±0.44 years), of whom 50.3% were females (Table 1).

**Table 1:** Demographic and socio-economic characteristics of the study respondents.

Variable	Frequency (n)	Percentage (%)
**Sex**		
Male	798	49.7
Female	807	50.3
**Age, x (SD)**	15.26	0.44
**Region**		
Belgrade	319	19.9
Vojvodina	496	30.9
Šumadija and Western Serbia	537	33.5
Southern and Eastern Serbia	253	15.8
**Type of school**		
Grammar school	345	21.5
Secondary vocational school	1260	78.5
**Socio-economic status of the family, x (SD)**	7.29	2.49

Approximately the same number of students expressed high satisfaction with school and high schoolwork pressure (43.8% and 43.4%, respectively) (Table 2). Less than half of students reported high teacher support (41.5%), while classmate support was high for approximately two-thirds of respondents (67.2%). Around 14% of students reported being bullied at school. The largest number of students expressed high level of life satisfaction (88.3%), but more than a quarter (26.1%) and more than a half (56.8%) reported symptoms of depression and psychosomatic health complaints, respectively.

**Table 2:** Psychosocial school and out-of-school environment and mental health of the study respondents.

Variable	Frequency (n)	Percentage (%)
**Satisfaction with school**		
Low	893	56.2
High	697	43.8
**Schoolwork pressure**		
Low	903	56.6
High	691	43.4
**Teacher support**		
Low	923	58.5
High	654	41.5
**Classmate support**		
Low	518	32.8
High	1060	67.2
**Bullying at school**		
No	1351	86.2
Yes	217	13.8
**Perceived friend support**		
Low	658	42.0
High	909	58.0
**Perceived family support**		
Low	314	20.2
High	1243	79.8
**Life satisfaction**		
Low	187	11.7
High	1405	88.3
**Symptoms of depression**		
No	1163	73.9
Yes	410	26.1
**Psychosomatic health complaints**		
No	683	43.2
Yes	897	56.8

There was a statistically significant difference in satisfaction with school, schoolwork pressure, teacher support and friend support among students who attended grammar school and secondary vocational school (S1 Table). Namely, satisfaction with school, schoolwork pressure and friend support were higher in grammar school students, while perceived teacher support was higher in secondary vocational school students.

The level of life satisfaction was significantly higher among male students and in students with higher socio-economic status (S2 Table). Also, there was a statistically significant difference in life satisfaction in relation to all observed psychosocial factors of the school and wider environment.

Table 3 shows the results of univariable and multivariable logistic regression with life satisfaction as the outcome variable. In univariable analysis, male students were 1.8 times more likely to have high life satisfaction, while for each unit increase on the FAS scale, the probability of high life satisfaction increased by 16.4%. Furthermore, life satisfaction was positively related to satisfaction with school, support from teachers and classmates, and negatively related to schoolwork pressure and being bullied at school. Also, there was a positive association between high life satisfaction and support from family and friends. In the multivariable analysis, in the last step of the backward analysis, male sex, higher perceived family socio-economic status, high satisfaction with school, high classmate support, not being bullied at school, high support of family and friends (the latter with marginal significance) were singled out as independent predictors of high life satisfaction.

**Table 3:** Logistic regression analysis of factors related to life satisfaction among students.

Characteristic	Univariable analysis		Multivariable analysis			
			Model 1 (enter) ^a^		Model 2 (backward, last step) ^b^	
	OR (95% CI)	p	OR (95% CI)	p	OR (95% CI)	p
**Sex (reference: male)**	0.555 (0.405–0.760)	<0.001*	0.611 (0.421–0.887)	0.010*	0.626 (0.436–0.900)	0.011*
**Region**						
Belgrade	reference					
Vojvodina	1.165 (0.750–1.808)	0.497				
Šumadija and Western Serbia	1.122 (0.730–1.727)	0.600				
Southern and Eastern Serbia	0.838 (0.515–1.363)	0.476				
**Type of school (reference: grammar school)**	0.699 (0.466–1.049)	0.083	0.726 (0.453–1.163)	0.183		
**Family socioeconomic status**	1.164 (1.091–1.242)	<0.001*	1.144 (1.063–1.231)	<0.001*	1.147 (1.068–1.231)	<0.001*
**Satisfaction with school (ref: Low)**	1.864 (1.344–2.587)	<0.001*	1.327 (0.890–1.977)	0.165	1.559 (1.077–2.256)	0.019*
**Schoolwork pressure (ref: Low)**	0.567 (0.417–0.771)	<0.001*	0.720 (0.495–1.048)	0.086		
**Teacher support (ref: Low)**	1.792 (1.281–2.506)	0.001*	1.216 (0.818–1.807)	0.334		
**Classmate support (ref: Low)**	2.527 (1.850–3.453)	<0.001*	1.464 (1.011–1.119)	0.043*	1.586 (1.106–2.274)	0.012*
**Being bullied at school (ref: No)**	0.352 (0.245–0.505)	<0.001*	0.550 (0.360–0.841)	0.006*	0.565 (0.370–0.861)	0.008*
**Friend support (ref: Low)**	1.939 (1.416–2.655)	<0.001*	1.456 (1.014–2.090)	0.042*	1.429 (0.998–2.044)	0.051
**Family support (ref: Low)**	4.685 (3.381–6.492)	<0.001*	3.654 (2.547–5.243)	<0.001*	3.794 (2.650–5.430)	<0.001*

^a^ Nagelkerke R ^2^ = 0.184

^b^ Nagelkerke R ^2^ = 0.178

OR–odds ratio, CI–confidence interval

*—statistical significance

On the other hand, symptoms of depression were significantly more common in girls, and there was also a statistically significant difference in the presence of symptoms of depression in relation to the region, all analyzed psychosocial factors of the school environment, support from family and friends (S3 Table).

Table 4 demonstrates the results of univariable and multivariable logistic regression with symptoms of depression as an outcome variable. Univariable analysis indicated that girls were 3.3 times more likely to have symptoms of depression compared to boys, and also students who attended school in Vojvodina and the region of Šumadija and Western Serbia had a 39% and 35% lower probability of symptoms of depression compared to the students of Belgrade schools, respectively. Type of school was at the borderline of significance, with the protective factor being attendance at a secondary vocational school versus grammar school. All psychosocial factors of the school environment were significantly related to depressive symptoms, with school satisfaction, teacher and classmate support acting protectively, and schoolwork pressure and being bullied at school being risk factors for depressive symptoms. In a multivariable analysis, students under high schoolwork pressure and those who were bullied at school were 2.4 and 2.2 times more likely to have depressive symptoms than those who were not, respectively. On the other hand, the support of the classmates and teachers was protective, as did the support of friends and family. Also, female sex was the biggest risk factor for depressive symptoms.

**Table 4:** Logistic regression analysis of factors associated with depressive symptoms among students.

Variable (reference group)	Univariable analysis		Multivariable analysis			
			Model 1 (enter) ^a^		Model 2 (backward, last step) ^b^	
	OR (95% CI)	p	OR (95% CI)	p	OR (95% CI)	p
**Sex (ref: Male)**	3.309 (2.593–4.222)	<0.001*	3.803 (2.832–5.107)	<0.001*	3.788 (2.838–5.054)	<0.001*
**Region**						
Belgrade	reference		reference			
Vojvodina	0.613 (0.447–0.842)	0.002*	0.848 (0.588–1.221)	0.374		
Šumadija and Western Serbia	0.655 (0.481–0.893)	0.007*	0.776 (0.542–1.110)	0.165		
Southern and Eastern Serbia	0.770 (0.534–1.109)	0.160	0.973 (0.634–1.494)	0.902		
**Type of school (ref: Grammar school)**	0.773 (0.593–1.008)	0.057	1.179 (0.853–1.630)	0.318		
**Family socioeconomic status**	0.973 (0.929–1.018)	0.234				
**Satisfaction with school (ref: Low)**	0.656 (0.519–0.828)	<0.001*	1.022 (0.764–1.367)	0.883		
**Schoolwork pressure (ref: Low)**	2.492 (1.978–3.141)	<0.001*	2.502 (1.875–3.337)	<0.001*	2.456 (1.879–3.210)	<0.001*
**Teacher support (ref: Low)**	0.450 (0.352–0.575)	<0.001*	1.216 (0.818–1.807)	0.334	0.704 (0.527–0.941)	0.018*
**Classmate support (ref: Low)**	0.389 (0.308–0.491)	<0.001*	0.708 (0.534–0.938)	0.016*	0.702 (0.532–0.927)	0.013*
**Being bullied at school (ref: No)**	3.010 (2.234–4.058)	<0.001*	2.205 (1.547–3.143)	<0.001*	2.166 (1.521–3.084)	<0.001*
**Friend support (ref: Low)**	0.591 (0.470–0.743)	<0.001*	0.627 (0.475–0.827)	0.001*	0.627 (0.475–0.826)	0.001*
**Family support (ref: Low)**	0.286 (0.220–0.372)	<0.001*	0.360 (0.265–0.489)	<0.001*	0.360 (0.265–0.488)	<0.001*

^**a**^ Nagelkerke R ^2^ = 0.268

^b^ Nagelkerke R ^2^ = 0.266

OR–odds ratio, CI–confidence interval

*—statistical significance

When it comes to the presence of psychosomatic health complaints, there was a statistically significant difference in relation to sex, region, type of school and all analyzed psychosocial factors of the school environment, as well as family support. Namely, girls and students from Belgrade schools and grammar schools reported psychosomatic health complaints significantly more often. They were also more common in students with low school satisfaction, high schoolwork pressure, low support from teachers and classmates, students who were bullied, and those who had low family support (S4 Table).

Table 5 shows the results of univariable and multivariable logistic regression analysis with the presence of psychosomatic complaints as an outcome variable. In the univariable analysis, female students were three times more likely to have multiple psychosomatic health complaints than male students, as well as grammar school students 1.4 times more likely than students of secondary vocational schools. Also, schoolwork pressure and being bullied at school were positively related, while satisfaction with school, support of teachers and classmates were negatively related to psychosomatic health complaints. Support of friends was marginally significant, while support of family had a protective effect on the presence of psychosomatic health complaints. As independent predictors in the last step of the multivariable analysis, female sex and all observed factors of the psychosocial school environment were singled out, with the most significant factor being schoolwork pressure. Also, family support was the most significant protective factor.

**Table 5:** Logistic regression analysis of factors associated with psychosomatic health complaints among students.

Variable (reference group)	Univariable analysis		Multivariable analysis			
			Model 1 (enter) ^a^		Model 2 (backward, last step) ^b^	
	OR (95% CI)	p	OR (95% CI)	p	OR (95% CI)	p
**Sex (ref: male)**	3.067 (2.493–3.773)	<0.001*	3.199 (2.519–4.063)	<0.001*	3.130 (2.482–3.948)	<0.001*
**Region**						
Belgrade	reference		reference			
Vojvodina	0.656 (0.491–0.876)	0.004*	0.853 (0.613–1.189)	0.349		
Šumadija and Western Serbia	0.747 (0.561–0.995)	0.046*	0.960 (0.694–1.329)	0.808		
Southern and Eastern Serbia	0.713 (0.508–0.999)	0.049*	0.864 (0.586–1.273)	0.460		
**Type of school (ref: grammar school)**	0.732 (0.572–0.935)	0.013*	1.047 (0.784–1.398)	0.757		
**Family socioeconomic status**	1.014 (0.973–1.055)	0.513				
**Satisfaction with school (ref: Low)**	0.516 (0.421–0.632)	<0.001*	0.733 (0.573–0.938)	0.013*	0.736 (0.577–0.938)	0.013*
**Schoolwork pressure (ref: Low)**	2.684 (2.174–3.314)	<0.001*	2.261 (1.758–2.908)	<0.001*	2.265 (1.774–2.893)	<0.001*
**Teacher support (ref: Low)**	0.485 (0.395–0.596)	<0.001*	0.768 (0.603–0.979)	0.033*	0.765 (0.601–0.972)	0.029*
**Classmate support (ref: Low)**	0.482 (0.386–0.602)	<0.001*	0.796 (0.610–1.038)	0.093	0.768 (0.593–0.996)	0.046*
**Being bullied at school (ref: No)**	1,943 (1.420–2.658)	<0.001*	1.543 (1.075–2.216)	0.019*	1.558 (1.086–2.235)	0.016*
**Friend support (ref: Low)**	0.819 (0.667–1.004)	0.055	0.882 (0.692–1.124)	0.310		
**Family support (ref: Low)**	0.398 (0.302–0.525)	<0.001*	0.489 (0.357–0.669)	<0.001*	0.471 (0.346–0.641)	<0.001*

^a^ Nagelkerke R ^2^ = 0.218

^b^ Nagelkerke R ^2^ = 0.216

OR–odds ratio, CI–confidence interval

*—statistical significance

## Discussion

In this paper, we examined the association between psychosocial school factors and mental health of the first grade secondary school students in Serbia. We found that certain school psychosocial school factors are associated with life satisfaction, depressive symptoms and psychosomatic health complaints, adjusted for demographic, socio- economic and social support variables outside the school environment.

In our study, life satisfaction was positively related to satisfaction with school and classmate support, and negatively to being bullied at school. This finding is mostly in line with previous research. Wahlström et al. also identified classmate support as an independent predictor of high life satisfaction in the population of Swedish adolescents, but also the support of teachers and high perceived school demands, which did not stand out as significant in our study in the multivariable analysis, although they were previously significant in the univariable analysis [6]. However, their study did not include other factors of the school environment, such as school satisfaction and bullying, and also did not analyze the social context in terms of family and friend support, which potentially attenuated the importance of schoolwork pressure and teacher support in our study. Teacher and classmate support has also been identified as a significant predictor of adolescent life satisfaction in Spain and Portugal [8] and in Slovenia [36]. Furthermore, a study in Greece also identified school satisfaction as an independent predictor of life satisfaction in 15-year-olds, along with schoolwork pressure, communication with parents and their support [7], but this study also did not include other factors that we considered. A study by Jovanovic and Jerkovic also indicated that students who are satisfied with school manifest greater general satisfaction with life [4]. Satisfaction with school also stood out as an independent predictor of life satisfaction in Hungarian middle and high school students [37]. Similar to our study, in most of the Nordic countries included in the study by Arnarsson et al., a significant association of being bullied with a lower level of life satisfaction was found [9], as well as in other European countries [10, 11], though observed independently of other school factors. In addition, a study among Croatian adolescents indicated that students in poor school environment (based on classmate support, academic achievement, liking school and school-related stress) had 4.3 times higher odds to be dissatisfied with life compared to those in favourable school environment [38].

In our study independent predictors of the presence of depressive symptoms were all observed psychosocial factors of the school environment, except for school satisfaction. In addition, the strongest predictors were schoolwork pressure and being bullied at school. This is consistent with a study among Norwegian adolescents, where stress related to school and bullying were also the most significant factors associated with symptoms of depression and anxiety [2]. Other studies also indicated the significance of the association between school-related stress and academic success and depressive symptoms [13, 39]. Bullying has also been described as a significant risk factor for depression [12, 15, 16]. Also, it has been described that a greater sense of school connectedness and a good relationship with teachers are associated with fewer symptoms of depression [14]. A longitudinal study that followed up 2465 adolescents in Norway indicated that teacher support was the most significant predictor at the beginning of the study and the only significant school predictor of depressive symptoms after one year [13]. Classmate support was also reported to be significant in the literature [24], though it was not significantly associated with depressive symptoms in the study by Dahlqvist et al. [25]. In the aforementioned study, there were also differences in the importance of psychosocial factors in relation to sex, where a greater number of these factors were significant in girls [25], which was not examined in our study. In our study there was a significant association between school satisfaction and depressive symptoms, but it was not confirmed in the multivariable analysis. Data from the literature on the association between satisfaction with school and depressive symptoms are scarce [4, 36]. In the existing literature, a significant association between school satisfaction and depression symptoms has been identified, although it is important to emphasize that school satisfaction is defined differently, e.g. as a composite variable that, in addition to the statement about liking the school, also includes the attitude of the teacher and other students towards the student, as well as schoolwork pressure [40]. According to a Hungarian study, being happy with school had a protective role in depressive symptomatology among boys [41]. In the regional context, it is important to mention that among Croatian adolescents school factors such as school attachment and school commitment were also associated with depression, though different methodology makes comparison chalenging [42].

In this paper, all analyzed factors of the school environment were significantly related to psychosomatic health complaints, whereby schoolwork pressure and bullying at school were positively related, while teacher and classmate support and satisfaction with school were negatively related. These results are in line with the previous literature. Namely, it is described that the psychosocial work environment at school is associated with psychological and somatic complaints [21, 38]. For instance, 15-year-old students with poor school environment in Croatia were 4.1 times more likely to have multiple psychosomatic health complaints compared to the ones with favourable school environment [38]. Furthermore, a study that comprised students from Canada, Norway and Romania demonstrated the association between school climate (defined by teacher support and school pressure) and psychosomatic health complaints despite cross-country educational and cultural differences [43]. Analogous to our results, among Greek 15-year-olds, psychosomatic health complaints were also associated with school workload and school satisfaction [7]. Other studies have also determined that schoolwork pressure is directly related to psychosomatic health complaints [18–20]. In a study conducted in Ireland, students who were bullied were more than twice as likely to have psychological and somatic complaints, while in our study this probability was slightly lower (OR = 1.56) [10]. Contrary to our results, in the study by Shaheen et al. there was no significant correlation between the support of classmates and psychosomatic health complaints [22]. In the same study, there was a significant association of other variables related to the school environment (school climate, school workload, teacher support, bullying) with psychosomatic health complaints, but it was of low strength. On the other hand, it was also described that acceptance by other students, one of the elements of classmate support, is negatively related to the score of psychosomatic health complaints [23], which coincides with our results.

The results of this study confirm the complexity of the relationship between psychosocial factors of the school environment with positive and negative aspects of mental health. Overall, most psychosocial factors of the school environment were significantly associated with both positive (life satisfaction) and negative aspects of mental health (symptoms of depression, psychosomatic health complaints). At the same time, the support of classmates and being bullied at school were significant independent predictors for all three observed parameters of mental health.

We consider these findings valuable to prepare evidence based health promoting intervention programmes aimed at improving mental health and wellbeing of Serbian adolescents. The newly adopted national Youth strategy for the period 2023–2030 foresees measures of support to programmes that contribute to mental health of young people [44]. The importance of improvement of mental health and wellbeing of this population is also reflected in the national Programme on mental health protection in the Republic of Serbia for the period 2019–2026, in which children and young people are identified as a sensitive population group that will be in focus of the future work in this area [45]. The Programme among other activities foresees establishing a network of multidisciplinary teams within mental health centres for children and young people, which would function in close cooperation with other institutions including schools. Based on the research findings, we believe that, within the work of professional teams, special attention should be paid to the psychosocial factors of the school environment as potentially contributing factors in the context of mental health.

### Strengths and limitations of the study

Our study had certain limitations: cross-sectional study design that prevents conclusions about the causality between the psychosocial school factors and mental health; not taking into account other factors that could be related to impaired mental health and wellbeing of students, such as objective health status of students and the presence of chronic diseases, or their academic success, or other possible stressors such as negative/ traumatic life events, family conflict etc. According to our knowledge, this is the first study in Serbia that systematically examines the relationship between psychosocial factors of the school environment and mental health of students on a nationally representative sample. It allows comparison with other countries from the HBSC network using the same methodology and study protocol.

Further studies should focus on the interactions of psychosocial school factors and also include other important factors in order to determine the magnitude of the contribution of factors of school environment. Also, in further research, the population of senior high school students could be included in order to observe the dynamics between psychosocial factors of the school environment and mental health during schooling. It would be of great importance to examine whether and how new teaching models, such as online teaching, have influenced this dynamic.

## Conclusions

The results of our research indicate that the psychosocial school factors were associated with mental health, specifically life satisfaction, symptoms of depression and psychosomatic health complaints among first grade secondary school students in Serbia.

Life satisfaction was positively related to satisfaction with school and classmate support, and negatively related to bullying at school.

Symptoms of depression were significantly related to all psychosocial factors of the school environment except satisfaction with school. There was a positive association of depressive symptoms with schoolwork pressure and being bullied at school, and a negative association with teacher and classmate support.

All analyzed factors of the school environment were significantly related to psychosomatic health complaints, whereby schoolwork pressure and being bullied at school were positively related, and the support of teachers and students and satisfaction with school were negatively related.

Bearing in mind the high prevalence of impaired mental health in the population of the first grade secondary school students and their significant association with the psychosocial school factors, as well as the fact that these factors are modifiable, it is necessary to apply appropriate measures at the school and community level with the aim of improving social support in the school environment, reducing the level of schoolwork burden and preventing abuse at school. In the future, this would potentially contribute to improvement of mental health of first grade students.

## Supporting information

S1 Table Psychosocial school and out-of-school factors among students by school type. (DOCX)

S2 Table Life satisfaction in relation to psychosocial school and other factors. (DOCX)

S3 Table Symptoms of depression in relation to psychosocial school and other factors. (DOCX)

S4 Table Psychosomatic health complaints in relation to psychosocial school and other factors. (DOCX)

S1 Checklist STROBE statement—Checklist of items that should be included in reports of observational studies. (DOCX)
