# Supplementary material for: Psychosocial school factors and mental health of first grade secondary school students—Results of the Health Behaviour in School-aged Children Survey in Serbia
Source: PLoS One. 2023 Nov 9;18(11):e0293179. doi: 10.1371/journal.pone.0293179 (PMC10635433; doi:10.1371/journal.pone.0293179)
Supplement: S1 Table — (DOCX) [file pone.0293179.s001.docx]

| **Characteristic** | **Grammar school**  **N=345 (21.5%)** | | | **Secondary Vocational School**  **N=1260 (78.5%)** | | **Test result** |
| --- | --- | --- | --- | --- | --- | --- |
|  | **n** | | **%** | **n** | **%** |  |
| **Satisfaction with school** | | | | | | |
| Low | 176 | 19.7 | | 717 | 80.3 | χ2= 3.912  p=0.049* |
| High | 166 | 23.8 | | 531 | 76.2 |  |
| **Schoolwork pressure** | | | | | | |
| Low | 142 | 15.7 | | 761 | 84.3 | χ2= 41.391  p=0.001* |
| High | 201 | 29.1 | | 490 | 70.9 |  |
| **Teacher support** | | | | | | |
| Low | 235 | 25.5 | | 688 | 74.5 | χ2= 18.662  p<0.001* |
| High | 107 | 16.4 | | 547 | 83.6 |  |
| **Classmate support** | | | | | | |
| Low | 98 | 18.9 | | 420 | 81.1 | χ2= 3.296  p=0.079 |
| High | 243 | 22.9 | | 817 | 77.1 |  |
| **Being bullied at school** | | | | | | |
| No | 291 | 21.5 | | 1060 | 78.5 | χ2= 0.248  p=0.658 |
| Yes | 50 | 23.0 | | 167 | 77.0 |  |
| **Friend support** | | | | | | |
| Low | 121 | 18.4 | | 537 | 81.6 | χ2= 7.849  p=0.005* |
| High | 221 | 24.3 | | 688 | 75.7 |  |
| **Family support** | | | | | | |
| Low | 64 | 20.4 | | 250 | 79.6 | χ2= 0.488  p=0.541 |
| High | 276 | 22.2 | | 967 | 77.8 |  |

**S1 Table. Psychosocial school and out-of-school factors among students by school type.**

* - statistical significance
