# Supplementary material for: Psychosocial school factors and mental health of first grade secondary school students—Results of the Health Behaviour in School-aged Children Survey in Serbia
Source: PLoS One. 2023 Nov 9;18(11):e0293179. doi: 10.1371/journal.pone.0293179 (PMC10635433; doi:10.1371/journal.pone.0293179)
Supplement: S2 Table — (DOCX) [file pone.0293179.s002.docx]

**S2 Table. Life satisfaction in relation to psychosocial school and other factors.**

| **Characteristic** | **Life satisfaction, n (%)** | | **Test result** |
| --- | --- | --- | --- |
|  | Low  187 (11.7) | High  1405 (88.3) |  |
| **Sex** | | | |
| Male | 69 (8.7) | 721 (91.3) | χ2=13.724  p<0.001 ^a*^ |
| Female | 118 (14.7) | 684 (85.3) |  |
| **Region** | | | |
| Belgrade | 39 (12.3) | 278 (87.7) | χ2=2.405  p=0.493 ^a^ |
| Vojvodina | 53 (10.8) | 440 (89.2) |  |
| Šumadija and Western Serbia | 59 (11.1) | 472 (88.9) |  |
| Southern and Eastern Serbia | 36 (14.3) | 215 (85.7) |  |
| **Type of school** | | | |
| Grammar school | 31 (9.1) | 311 (90.9) | χ2=3.022  p=0.088 ^a^ |
| Secondary Vocational School | 156 (12.5) | 1094 (87.5) |  |
| **Material condition of the family, x (SD)** | 6.50 (2.67) | 7.40 (2.44) | t=-4.667  p<0.001 ^b*^ |
| **Satisfaction with school** | | | |
| Low | 128 (14.5) | 754 (85.5) | χ2=14.211  p<0.001 ^a*^ |
| High | 58 (8.3) | 637 (91.7) |  |
| **Schoolwork pressure** | | | |
| Low | 83 (9.2) | 815 (90.8) | χ2=13.321  p<0.001 ^a*^ |
| High | 104 (15.2) | 579 (84.8) |  |
| **Teacher support** | | | |
| Low | 128 (14.0) | 787 (86.0) | χ2=11.865  p=0.001 ^a*^ |
| High | 54 (8.3) | 595 (91.7) |  |
| **Classmate support** | | | |
| Low | 96 (18.6) | 420 (81.4) | χ2=35.611  p<0.001 ^a*^ |
| High | 87 (8.3) | 962 (91.7) |  |
| **Bullying at school** | | | |
| No | 133 (9.9) | 1209 (90.1) | χ2=34.305  p<0.001 ^a*^ |
| Yes | 51 (23.8) | 163 (76.2) |  |
| **Support from friends** | | | |
| Low | 101 (15.5) | 551 (84.5) | χ2=17.456  p<0.001 ^a*^ |
| High | 78 (8.6) | 825 (91.4) |  |
| **Family support** | | | |
| Low | 86 (27.7) | 225 (72.3) | χ2=97.998  p<0.001 ^a*^ |
| High | 93 (7.5) | 1140 (92.5) |  |

^a^ Chi square test, ^b^ two-tailed t test, * - statistical significance
