# Supplementary material for: Psychosocial school factors and mental health of first grade secondary school students—Results of the Health Behaviour in School-aged Children Survey in Serbia
Source: PLoS One. 2023 Nov 9;18(11):e0293179. doi: 10.1371/journal.pone.0293179 (PMC10635433; doi:10.1371/journal.pone.0293179)
Supplement: S3 Table — (DOCX) [file pone.0293179.s003.docx]

**S3 Table. Symptoms of depression in relation to psychosocial school and other factors.**

| **Characteristic** | **Symptoms of depression, n (%)** | | **Test result** |
| --- | --- | --- | --- |
|  | Yes  410 (26.1) | No  1163 (73.9) |  |
| **Gender** | | | |
| Male | 117 (15.0) | 662 (85.0) | χ2=97.705  p<0.001 ^a*^ |
| Female | 293 (36.9) | 501 (63.1) |  |
| **Region** | | | |
| Belgrade | 103 (32.8) | 211 (67.2) |  |
| Vojvodina | 112 (23.0) | 374 (77.0) | χ2=10.805 |
| Šumadija and Western Serbia | 127 (24.2) | 397 (77.0) | p=0.013 ^a*^ |
| Southern and Eastern Serbia | 68 (27.3) | 181 (72.7) |  |
| **Type of school** | | | |
| Grammar school | 102 (30.1) | 237 (69.9) | χ2=3.630  p=0.060 ^a^ |
| Secondary Vocational School | 308 (25.0) | 926 (75.0) |  |
| **Material condition of the family, x (SD)** | 7.18 (2.61) | 7.35 (2.44) | t=1.190  p=0.234 ^b^ |
| **Satisfaction with school** | | | |
| Low | 257 (29.4) | 617 (70.6) | χ2=12.627  p=0.001 ^a*^ |
| High | 147 (21.5) | 538 (78.5) |  |
| **Schoolwork pressure** | | | |
| Low | 164 (18.4) | 725 (81.6) | χ2=61.699  p <0.001 ^a*^ |
| High | 243 (36.1) | 431 (63.9) |  |
| **Teacher support** | | | |
| Low | 292 (32.1) | 617 (67.9) | χ2=41.547  p<0.001 ^a*^ |
| High | 113 (17.5) | 531 (82.5) |  |
| **Classmate support** | | | |
| Low | 200 (38.7) | 317 (61.3) | χ2=64.645  p<0.001 ^a*^ |
| High | 204 (19.7) | 832 (80.3) |  |
| **Bullying at school** | | | |
| Not | 304 (22.9) | 1025 (77.1) | χ2=55.792  p <0.001 ^a*^ |
| Yes | 100 (47.2) | 112 (52.8) |  |
| **Support from friends** | | | |
| Low | 101 (15.5) | 551 (84.5) | χ2=20.501  p <0.001 ^a*^ |
| High | 78 (8.6) | 825 (91.4) |  |
| **Family support** | | | |
| Low | 147 (47.9) | 160 (52.1) | χ2=93.087  p <0.001 ^a*^ |
| High | 255 (20.8) | 971 (79.2) |  |

^a^ Chi square test, ^b^ two-tailed t test, * - statistical significance
