# Supplementary material for: Psychosocial school factors and mental health of first grade secondary school students—Results of the Health Behaviour in School-aged Children Survey in Serbia
Source: PLoS One. 2023 Nov 9;18(11):e0293179. doi: 10.1371/journal.pone.0293179 (PMC10635433; doi:10.1371/journal.pone.0293179)
Supplement: S4 Table — (DOCX) [file pone.0293179.s004.docx]

**S4 Table. Psychosomatic health complaints in relation to psychosocial school and other factors.**

| **Characteristic** | **Psychosomatic health complaints, n (%)** | | **Test result** |
| --- | --- | --- | --- |
|  | Yes  897 (56.8) | No  683 (43.2) |  |
| **Sex** | | | |
| Male | 337 (43.2) | 443 (56.8) | χ2=115.540  p<0.001 ^a*^ |
| Female | 560 (70.0) | 240 (30.0) |  |
| **Region** | | | |
| Belgrade | 202 (63.5) | 116 (36.5) | χ2=8.483  p=0.037 ^a*^ |
| Vojvodina | 258 (53.3) | 226 (46.7) |  |
| Šumadija and Western Serbia | 298 (56.5) | 229 (43.5) |  |
| Southern and Eastern Serbia | 139 (55.4) | 112 (44.6) |  |
| **Type of school** | | | |
| Grammar school | 215 (62.7) | 128 (37.3) | χ2=6.235  p=0.014 ^a*^ |
| Secondary Vocational School | 682 (55.1) | 555 (44.9) |  |
| **Family affluence** | 7.34 (2.54) | 7.26 (2.40) | t = -0.653  p=0.514 ^b^ |
| **Satisfaction with school** | | | |
| Low | 563 (63.9) | 318 (36.1) | χ2=41.059  p<0.001 ^a*^ |
| High | 327 (47.7) | 358 (52.3) |  |
| **Schoolwork pressure** | | | |
| Low | 417 (46.7) | 475 (53.3) | χ2=86.421  p<0.001 ^a*^ |
| High | 476 (70.2) | 202 (29.8) |  |
| **Teacher support** | | | |
| Low | 589 (64.3) | 327 (35.7) | χ2=47.969  p<0.001 ^a*^ |
| High | 299 (46.6) | 342 (53.4) |  |
| **Classmate support** | | | |
| Low | 351 (68.6) | 161 (31.4) | χ2=42.016  p<0.001 ^a*^ |
| High | 536 (51.2) | 510 (48.8) |  |
| **Being bullied at school** | | | |
| Not | 734 (55.1) | 599 (44.9) | χ2=17.694  p<0.001 ^a*^ |
| Yes | 150 (70.4) | 63 (29.6) |  |
| **Support from friends** | | | |
| Low | 389 (59.8) | 262 (40.2) | χ2=3.681  p=0.061 ^a^ |
| High | 491 (54.9) | 404 (45.1) |  |
| **Family support** | | | |
| Low | 230 (73.7) | 82 (26.3) | χ2=44.488  p<0.001 ^a*^ |
| High | 646 (52.8) | 578 (47.2) |  |

^a^ Chi square test, ^b^ two-tailed t test, * - statistical significance
