# Supplementary material for: Psychosocial school factors and mental health of first grade secondary school students—Results of the Health Behaviour in School-aged Children Survey in Serbia
Source: PLoS One. 2023 Nov 9;18(11):e0293179. doi: 10.1371/journal.pone.0293179 (PMC10635433; doi:10.1371/journal.pone.0293179)
Supplement: S1 Checklist — (DOCX) [file pone.0293179.s005.docx]

STROBE Statement—checklist of items that should be included in reports of observational studies

|  | Item No. | Recommendation | Page  No. | Relevant text from manuscript |
| --- | --- | --- | --- | --- |
| **Title and abstract** | 1 | (*a*) Indicate the study’s design with a commonly used term in the title or the abstract | 2 | This cross-sectional study aimed to investigate the association between psychosocial school factors and life satisfaction, symptoms of depression and psychosomatic health complaints among first grade secondary school students in Serbia. |
|  |  | (*b*) Provide in the abstract an informative and balanced summary of what was done and what was found | 2,3 | This cross-sectional study aimed to investigate the association between psychosocial school factors and life satisfaction, symptoms of depression and psychosomatic health complaints among first grade secondary school students in Serbia. We analysed data from the 2018 Health Behaviour in School-aged Children (HBSC) study in the Republic of Serbia. Analyzed psychosocial school factors included satisfaction with school, schoolwork pressure, teacher support, classmate support and being bullied at school. Life satisfaction was assessed by the 11-step Cantril’s ladder (cutoff >5). Symptoms of depression were measured by the Center for Epidemiologic Studies Depression Scale (CESD-10) and psychosomatic heath complaints by using the HBSC symptom checklist. Univariable and multivariable binary logistic regression was used to determine independent predictors of students’ life satisfaction, symptoms of depression and psychosomatic health complaints in the school environment, while also considering their socio-demographic characteristics and perceived family and friend support. The study included 1605 students (average age 15.26 ±0.44 years), of whom 50.3% were females. Results from the binary logistic regression analyses showed that life satisfaction was positively related to school satisfaction and classmate support, and negatively to being bullied at school. Symptoms of depression were positively associated with schoolwork pressure and being bullied at school, and negatively with teacher and classmate support. All analyzed factors of the school environment were significantly related to psychosomatic health complaints, whereby schoolwork pressure and being bullied at school were positively associated, while teacher and classmate support and satisfaction with school were negatively associated. Given the established association of psychosocial school factors with mental health, there is a need for targeted measures both at school and community level with the aim of improving social support in the school environment, reducing schoolwork burden and preventing bullying at school, potentially resulting in the overall improvement of mental health of the first grade secondary school students. |
| Introduction | | | |  |
| Background/rationale | 2 | Explain the scientific background and rationale for the investigation being reported | 4, 5 | Schools represent an environment in which students spend a significant part of their time during childhood and adolescence. A supportive school environment can contribute to the development of healthy lifestyles and good mental health of students [1].  The definition of mental health has changed over time from negative aspects, through positive, to an integrative concept, where it is described as a continuum that moves from mental problems to well-being, or is viewed from multiple dimensions [2]. Namely, the same person can simultaneously have a high level of well-being, but also mental problems, so it is necessary to look at mental health in the widest possible context. The World Health Organisation (WHO) also emphasizes that people with a mental health disorder are more likely to have a low level of mental well-being, but this is not and may not always be the case [3]. In this sense, Rindgal et al. state the importance of examining the effects of potential predictors in identical models for both positive and negative aspects of mental health [2].  Life satisfaction represents an important aspect of subjective well-being, one of the most important components in modern models of mental health [4]. It is defined as "a general assessment that people make when considering their life as a whole" [5]. It is believed that the level of satisfaction with life can affect future health and even mortality [6]. There are numerous studies dealing with the determinants of life satisfaction, including those at the individual and family, school, community and national level. In the school environment, the literature data include the importance of satisfaction with school [4,7], perceived schoolwork pressure, teacher [6,8] and classmate support [6,8], as well as being bullied at school [9–11].  Depression is one of the most common mental health disorders in adolescence with an estimated prevalence of 4-5% in middle and late adolescence [12]. In the school environment, factors related to depressive symptoms or to occurrence of depression in adolescence include stress related to school and academic success [2,13], school connectedness [14], relationship with teachers [13,14] and bullying [2,12,15,16].  Estimated prevalence of psychosomatic health complaints in children and adolescents ranges between 10% and 25% [17]. Clinical manifestations in adolescents may be different when compared to adults. The most common symptoms in children are abdominal pain, headache, chest pain, fatigue, back pain, and breathing difficulties [17]. Symptoms vary and usually two or more occur at the same time and persist for longer periods of time. Previous research identified perceived schoolwork pressure, school satisfaction (including teacher and peer support) and bullying as factors in the school environment contributing to occurrence of psychosomatic health complaints [7,10,18–23].  Results from former research studies vary regarding the importance and influence of different factors in the school environment on mental health. For instance, some studies report that classmate support is significantly associated with psychosomatic health complaints [19,21], while some did not find this association significant [22]. There are also different findings regarding the significance of classmate support in relation to symptoms of depression [24,25]. Additionally, previous studies often do not include both positive and negative aspects of mental health. Literature review revealed only few studies that examined relationship between broader number of psychosocial factors, including bullying at school, with occurrence of psychosomatic health complaints [26], depression [2], and life satisfaction. Moreover, the effect of protective factors such as family or friend support was not included. In Serbia according to our knowledge there are no studies on association between psychosocial factors in the school environment and mental health. |
| Objectives | 3 | State specific objectives, including any prespecified hypotheses | 5, 6 | Therefore, the aim of the current study was to examine the association of psychosocial factors in the school environment with life satisfaction, psychosomatic health complaints and symptoms of depression in the first grade secondary school students in Serbia. We hypothesized that there is an association between psychosocial factors of the school environment with these mental health parameters. |
| Methods | | | |  |
| Study design | 4 | Present key elements of study design early in the paper | 7-9 | This cross-sectional study presents a secondary analysis of data obtained from the Health Behavior in School-aged Children (HBSC) Study in Serbia in 2018. The national survey was carried out using the International protocol of the HBSC study survey protocol, in line with the WHO methodology described in more detail elsewhere [27,28]. According to the protocol, study participants were 11, 13 and 15 year old students. The participants were recruited from the selected sample of schools who accepted to participate in the survey. Sampling frame was list of primary and secondary schools in Serbia. A stratified, multistage sampling approach was used for the selection of the survey sample. Independent samples were selected for each of the age groups. Estimated average number of students per class was 20. Sample included 64 school for each grade, i.e. age group - 39 schools per each age group was selected as main sample and additional 25 replacement schools due to possibility that some students could refuse to fill in the questionnaire or that parents (or guardians) would not give their consent for children’s participation in the survey. After the initial school selection, list of classes for the defined age groups was made and using table of random numbers two classes for each age group were selected.  After sampling, selected schools were contacted first by mail informing them about the survey and asking their consent to participate in the survey. Replacements schools were included in case that school refused to participate, however replacement schools were selected so that they would resemble schools in original sample (according to size - number of students, region, etc.). In recruitment stage, after initial mail, schools were contacted by phone. Recruitment was done during March 2018. Data collection was done between April 16th and June 7th 2018 in selected schools during one school class.  …  In the HBSC study in Serbia in 2018, students enrolled in the fifth and seventh grade of the primary school and students in the first grade of secondary schools (according to school system in the Republic of Serbia) in selected schools and selected classes were provided information about the survey including anonymity and voluntary participation. Students with parental (or guardian) consent of selected classes who accepted to participate in the survey were eligible survey participants. There were 192 initially selected and 101 participating schools. Out of 384 classes, 200 participated. Out of 7680 students, 4028 participated. Refusals were either due to not having parental (or guardian) consent or due to absence because of illness on the day of survey. |
| Setting | 5 | Describe the setting, locations, and relevant dates, including periods of recruitment, exposure, follow-up, and data collection | 7 | Recruitment was done during March 2018. Data collection was done between April 16th and June 7th 2018 in selected schools during one school class. |
| Participants | 6 | (*a*) *Cohort study*—Give the eligibility criteria, and the sources and methods of selection of participants. Describe methods of follow-up  *Case-control study*—Give the eligibility criteria, and the sources and methods of case ascertainment and control selection. Give the rationale for the choice of cases and controls  *Cross-sectional study*—Give the eligibility criteria, and the sources and methods of selection of participants | 8;9 | Students with parental (or guardian) consent of selected classes who accepted to participate in the survey were eligible survey participants.  …  Target population in this study included first grade secondary school students, since we were particularly interested in the importance of psychosocial factors after the transition from elementary primary school to secondary school. Being a student of the first grade in secondary school was the only inclusion criterion and there were no exclusion criteria. |
|  |  | (*b*) *Cohort study*—For matched studies, give matching criteria and number of exposed and unexposed  *Case-control study*—For matched studies, give matching criteria and the number of controls per case |  |  |
| Variables | 7 | Clearly define all outcomes, exposures, predictors, potential confounders, and effect modifiers. Give diagnostic criteria, if applicable | 2; 13 | Analyzed psychosocial school factors included satisfaction with school, schoolwork pressure, teacher support, classmate support and being bullied at school.  …  In addition, univariable and multivariable binary logistic regression was used to determine the association of psychosocial factors of the school environment with life satisfaction, depressive symptoms, and psychosomatic complaints. The outcome variables were: life satisfaction (high/low), psychosomatic health complaints (yes/no) and depressive symptoms (yes/no). Apart from psychosocial school factors, we also included demographic and socio-economic characteristics of the study participants, as well as family and friend support into regression analyses due to their previously reported significance in the literature. |
| Data sources/ measurement | 8* | For each variable of interest, give sources of data and details of methods of assessment (measurement). Describe comparability of assessment methods if there is more than one group | 9-13 | School type was differentiated based on whether the student attended grammar school or secondary vocational school.  Four statistical regions were identified: Belgrade, Vojvodina, Šumadija and Western Serbia, Southern and Eastern Serbia.  Socio-economic status was determined based on the Family Affluence Scale (FAS III), a composite indicator which is used to measure objective socio-economic status within the HBSC study [29]. It is determined based on the following six questions: „How many computers does your family own“ (none – 0; one – 1; two – 2; more than two - 3), „Does your family own a car, a van or a truck“ (no – 0; yes, one – 1; yes, two or more - 2), „Do you have your own bedroom for yourself“ (no-1; yes-2), „Does your family have a dishwasher at home“ (no – 0; yes -1), „How many bathrooms (room with a bath/shower or both) are in your home“ (none – 0; one – 1; two – 2; more than two -3), „How many times did you and your family travel for vacation/holiday outside of Serbia in the last 12 months“ (not at all – 0; once – 1; twice – 2; more than twice - 3). By summing up these points, the score of the perceived family's socio-economic status was calculated (ranging from 0 to 13) and this variable was further analyzed as numerical.  School satisfaction was estimated based on the question: „How do you like school at present“ with answer options ranging from 1 – ”I like it a lot” (highest level of school satisfaction) to 4 - ”I don’t like it at all” (lowest level of school satisfaction). This variable was dichotomized so that the answers “I like it very much” and “I like it a bit” denote school satisfaction and the answers “I don’t like it very much” and “I don’t like it at all” school dissatisfaction.  Schoolwork pressure was determined by the question “How pressured do you feel by the schoolwork you have to do?” Answer options range from 1 –Not at all (lowest level of schoolwork pressure) to 4-A lot (highest level of schoolwork pressure). Answers were grouped so that a student was considered to be pressured by schoolwork if the answer was "Yes, a lot" and "Yes, very much", as opposed to "Yes, a little" and "Not at all".  Classmate support was determined based on answers from a 5-point Likert scale of agreement (from "strongly disagree" to "strongly agree") with the following statements: "Students in my class like to be together", "Most students in my class are friendly and want to help", "Other students accept me as I am". A sum-score was generated from the responses to the above three items ranging from 3 to 15. The final score was classified as high or low classmate support based on a cut-off value of ≥2.5 [30].  Teacher support was determined based on answers from a 5-point Likert scale of agreement (from "strongly disagree" to "strongly agree") with the following statements: "I feel that the teachers accept me as I am", "I feel that the teachers (professors) take care of me as a person", "I have great confidence in my teachers". The same scoring system was applied as for classmate support [30].  Being bullied at school (bullying victimization) was estimated by the question "How often have you been bullied at school in the past couple of months" with the possible answers “I have not been bullied at school in the past couple of months“, “ It has happened once or twice”, “2 or 3 times a month”, “About once a week” and “Several times a week”. Responses were grouped and dichotomized as „not bullied“ (if answered I have not been bullied) and „bullied“ (if answered all other answer options).  Life satisfaction was determined based on Cantrill's scale: "Here is a picture of a ladder. The top of the ladder “10” is the best possible life for you and the bottom “0” is the worst possible life for you. In general, where on the ladder do you feel you stand at the moment?”, where respondents indicated their life satisfaction on a scale from zero to 19. Cut-off value of ≥6 was used for categorization of respondents with high and low life satisfaction [31].  The presence of depressive symptoms was evaluated by using Short depression scale of the Center for Epidemiologic Studies Depression Scale (CESD-10) [32], which consists of 10 statements about behaviours or feelings: „I was bothered by things that usually don't bother me”, “I had trouble keeping my mind on what I was doing”, “I felt depressed”, “I felt that everything I did was an effort”, “I felt hopeful about the future”, “I felt fearful”, “My sleep was restless”, “I was happy”, “I felt lonely”, “I could not "get going”." Available answer categories were as follows: “Rarely or none of the time (less than 1 day)“, “Some or a little of the time (1-2 days)”, “Occasionally or a moderate amount of time (3-4 days)”,“All of the time (5-7 days)”. The total score is calculated by summing up the 10 items. A score of 10 or higher out of 30 is the cut-off for clinically significant depressive symptoms. Based on this value, a dichotomization was made into subjects with and without symptoms of depression.  Psychosomatic health complaints were determined using the HBSC symptom checklist, which includes the frequency of the following complaints in the last six months: headache, stomachache, backache, feeling low, irritability or bad temper, feeling nervous, difficulties in getting to sleep, feeling dizzy. Possible answers were: „About every day”, “More than once a week”, “About every week”, “About every month”, “Rarely or never”. Students who had two or more symptoms (complaints) at the same time at least once a week were classified as having psychosomatic health complaints [18].  Perceived family and friend support was evaluated based on two subscales constructed of the Multidimensional Scale of Perceived Social Support (MSPSS). Items on family support measure the perceived availability of emotional support and help within the family and respondents stated agreement with the statements on a 7-point Likert scale, ranging from „very strongly disagree" to "very strongly agree". The statements were as follows: "My family really tries to help me", " I get the emotional help and support I need from my family", " I can talk about my problems with my family", " My family is willing to help me make decisions". First the sum of responses was calculated, then its mean value. A cut-off score value of ≥5.5 points [30,33] was considered as high support.  Perceived friend support was assessed the same as perceived family support by using a 7- point Likert response scale. The statements were: "My friends really try to help me", "I can count on my friends when things go wrong“, "I have friends with whom I can share my joys and sorrows", " I can talk about my problems with my friends". A cut-off score of 5.5 points [33] was considered as high support.  Missing data for one or more statements for assessing classmate and teacher support, and friend and family support was considered as a missing value. Furthermore, missing data for one or more of the eight listed health complaints was considered as a missing value. Regarding the symptoms of depression, observations with two or more missing responses were not included in the analysis, while in the case of one missing response, that response was replaced by the mean value [34]. |
| Bias | 9 | Describe any efforts to address potential sources of bias | 7 | Replacements schools were included in case that school refused to participate, however replacement schools were selected so that they would resemble schools in original sample (according to size - number of students, region, etc.) |
| Study size | 10 | Explain how the study size was arrived at | 7; 8; 9; 15 | The national survey was carried out using the International protocol of the HBSC study survey protocol, in line with the WHO methodology described in more detail elsewhere [27,28]. According to the protocol, study participants were 11, 13 and 15-year-old students. The participants were recruited from the selected sample of schools who accepted to participate in the survey. Sampling frame was list of primary and secondary schools in Serbia. A stratified, multistage sampling approach was used for the selection of the survey sample. Independent samples were selected for each of the age groups. Estimated average number of students per class was 20. Sample included 64 school for each grade, i.e. age group - 39 schools per each age group was selected as main sample and additional 25 replacement schools due to possibility that some students could refuse to fill in the questionnaire or that parents (or guardians) would not give their consent for children’s participation in the survey. After the initial school selection, list of classes for the defined age groups was made and using table of random numbers two classes for each age group were selected.  …  There were 192 initially selected and 101 participating schools. Out of 384 classes, 200 participated. Out of 7680 students, 4028 participated. Refusals were either due to not having parental (or guardian) consent or due to absence because of illness on the day of survey.  …  Target population in this study included first grade secondary school students, since we were particularly interested in the importance of psychosocial factors after the transition from elementary primary school to secondary school.  …  In this study, 1605 students were included (average age 15.26 ±0.44 years), of whom 50.3% (807/1605) were females |

Continued on next page

| Quantitative variables | 11 | Explain how quantitative variables were handled in the analyses. If applicable, describe which groupings were chosen and why | 9-10 | Socio-economic status was determined based on the Family Affluence Scale (FAS III), a composite indicator which is used to measure objective socio-economic status within the HBSC study [29]. It is determined based on the following six questions: „How many computers does your family own“ (none – 0; one – 1; two – 2; more than two - 3), „Does your family own a car, a van or a truck“ (no – 0; yes, one – 1; yes, two or more - 2), „Do you have your own bedroom for yourself“ (no-1; yes-2), „Does your family have a dishwasher at home“ (no – 0; yes -1), „How many bathrooms (room with a bath/shower or both) are in your home“ (none – 0; one – 1; two – 2; more than two -3), „How many times did you and your family travel for vacation/holiday outside of Serbia in the last 12 months“ (not at all – 0; once – 1; twice – 2; more than twice - 3). By summing up these points, the score of the perceived family's socio-economic status was calculated (ranging from 0 to 13) and this variable was further analyzed as numerical. |
| --- | --- | --- | --- | --- |
| Statistical methods | 12 | (*a*) Describe all statistical methods, including those used to control for confounding | 13 | Categorical variables were presented as numbers and percentages, while continuous variables were presented as means and standard deviations. Chi square test and the two tailed t test were used to test the difference in life satisfaction, the frequency of psychosomatic health complaints and depressive symptoms in relation to the observed factors of the school environment, demographic and socio-economic factors. Also, the chi-square test was used to test the difference in psychosocial factors in relation to the type of school. In addition, univariable and multivariable binary logistic regression was used to determine the association of psychosocial factors of the school environment with life satisfaction, depressive symptoms, and psychosomatic complaints. The outcome variables were: life satisfaction (high/low), psychosomatic health complaints (yes/no) and depressive symptoms (yes/no). Variance inflation factor - VIF value of 5 was used as a criterion for examining collinearity of independent variables. Independent variables that were significant at the p value of <0.1 in the univariable analysis were included in the models using the enter and backward methods. A p value of <0.05 was used as the minimum level of significance throughout the analysis. |
|  |  | (*b*) Describe any methods used to examine subgroups and interactions | 13 | Also, the chi-square test was used to test the difference in psychosocial factors in relation to the type of school. |
|  |  | (*c*) Explain how missing data were addressed | 13 | Missing data for one or more statements for assessing classmate and teacher support, and friend and family support was considered as a missing value. Furthermore, missing data for one or more of the eight listed health complaints was considered as a missing value. Regarding the symptoms of depression, observations with two or more missing responses were not included in the analysis, while in the case of one missing response, that response was replaced by the mean value [34]. |
|  |  | (*d*) *Cohort study*—If applicable, explain how loss to follow-up was addressed  *Case-control study*—If applicable, explain how matching of cases and controls was addressed  *Cross-sectional study*—If applicable, describe analytical methods taking account of sampling strategy |  | NA |
|  |  | (*e*) Describe any sensitivity analyses |  | NA |
| Results | | | | |
| Participants | 13* | (a) Report numbers of individuals at each stage of study—eg numbers potentially eligible, examined for eligibility, confirmed eligible, included in the study, completing follow-up, and analysed | 8; 15 | Out of 7680 students, 4028 participated. *(refers to the HBSC study in Serbia)*  *…*  In this study, 1605 students were included (average age 15.26 ±0.44 years), of whom 50.3% (807/1605) were females (Table 1). |
|  |  | (b) Give reasons for non-participation at each stage | 8 | Refusals were either due to not having parental (or guardian) consent or due to absence because of illness on the day of survey. |
|  |  | (c) Consider use of a flow diagram |  |  |
| Descriptive data | 14* | (a) Give characteristics of study participants (eg demographic, clinical, social) and information on exposures and potential confounders | 15 | In this study, 1605 students were included (average age 15.26 ±0.44 years), of whom 50.3% (807/1605) were females (Table 1). Approximately the same number of students expressed high satisfaction with school and high schoolwork pressure (43.8% (697/1590) and 43.4% (691/1594), respectively). Less than half of students reported high teacher support (41.5%, 654/1577), while classmate support was high for approximately two-thirds of respondents (67.2%, 1060/1578). Around 14% (217/1568) of students reported being bullied at school. |
|  |  | (b) Indicate number of participants with missing data for each variable of interest | 15-17 | In this study, 1605 students were included (average age 15.26 ±0.44 years), of whom 50.3% (807/1605) were females (Table 1). Approximately the same number of students expressed high satisfaction with school and high schoolwork pressure (43.8% (697/1590) and 43.4% (691/1594), respectively). Less than half of students reported high teacher support (41.5%, 654/1577), while classmate support was high for approximately two-thirds of respondents (67.2%, 1060/1578). Around 14% (217/1568) of students reported being bullied at school. The largest number of students expressed high level of life satisfaction (88.3%, 1405/1592), but more than a quarter (26.1%, 410/1573) and more than a half (56.8%, 897/1580) reported symptoms of depression and psychosomatic health complaints, respectively.  *It is also demonstrated in Table 1, because not all numbers add up to the total number of participants (1605).* |
|  |  | (c) *Cohort study*—Summarise follow-up time (eg, average and total amount) |  |  |
| Outcome data | 15* | *Cohort study*—Report numbers of outcome events or summary measures over time |  |  |
|  |  | *Case-control study—*Report numbers in each exposure category, or summary measures of exposure |  |  |
|  |  | *Cross-sectional study—*Report numbers of outcome events or summary measures | 15 | The largest number of students expressed high level of life satisfaction (88.3%, 1405/1592), but more than a quarter (26.1%, 410/1573) and more than a half (56.8%, 897/1580) reported symptoms of depression and psychosomatic health complaints, respectively. |
| Main results | 16 | (*a*) Give unadjusted estimates and, if applicable, confounder-adjusted estimates and their precision (eg, 95% confidence interval). Make clear which confounders were adjusted for and why they were included | 17-19;  19-21;  21-23 | Table 2 shows the results of univariable and multivariable logistic regression with life satisfaction as the outcome variable. In univariable analysis, male students were 1.8 times more likely to have high life satisfaction, while for each unit increase on the FAS scale, the probability of high life satisfaction increased by 16.4%. Furthermore, life satisfaction was positively related to satisfaction with school, support from teachers and classmates, and negatively related to schoolwork pressure and being bullied at school. Also, there was a positive association between high life satisfaction and support from family and friends. In the multivariable analysis, in the last step of the backward analysis, male sex, higher perceived family socio-economic status, high satisfaction with school, high classmate support, not being bullied at school, high support of family and friends (the latter with marginal significance) were singled out as independent predictors of high life satisfaction.  …  Table 3 demonstrates the results of univariable and multivariable logistic regression with symptoms of depression as an outcome variable. Univariable analysis indicated that girls were 3.3 times more likely to have symptoms of depression compared to boys, and also students who attended school in Vojvodina and the region of Šumadija and Western Serbia had a 39% and 35% lower probability of symptoms of depression compared to the students of Belgrade schools, respectively. Type of school was at the borderline of significance, with the protective factor being attendance at a secondary vocational school versus grammar school. All psychosocial factors of the school environment were significantly related to depressive symptoms, with school satisfaction, teacher and classmate support acting protectively, and schoolwork pressure and being bullied at school being risk factors for depressive symptoms. In a multivariable analysis, students under high schoolwork pressure and those who were bullied at school were 2.4 and 2.2 times more likely to have depressive symptoms than those who were not, respectively. On the other hand, the support of the classmates and teachers was protective, as did the support of friends and family. Also, female sex was the biggest risk factor for depressive symptoms.  …  Table 4 shows the results of univariable and multivariable logistic regression analysis with the presence of psychosomatic complaints as an outcome variable. In the univariable analysis, female students were three times more likely to have multiple psychosomatic health complaints than male students, as well as grammar school students 1.4 times more likely than students of secondary vocational schools. Also, schoolwork pressure and being bullied at school were positively related, while satisfaction with school, support of teachers and classmates were negatively related to psychosomatic health complaints. Support of friends was marginally significant, while support of family had a protective effect on the presence of psychosomatic health complaints. As independent predictors in the last step of the multivariable analysis, female sex and all observed factors of the psychosocial school environment were singled out, with the most significant factor being schoolwork pressure. Also, family support was the most significant protective factor. |
|  |  | (*b*) Report category boundaries when continuous variables were categorized |  | NA |
|  |  | (*c*) If relevant, consider translating estimates of relative risk into absolute risk for a meaningful time period |  |  |

Continued on next page

| Other analyses | 17 | Report other analyses done—eg analyses of subgroups and interactions, and sensitivity analyses | 17 | There was a statistically significant difference in satisfaction with school (p=0.049, χ2=3.912), schoolwork pressure (p=0.001, χ2=41.391), teacher support (p<0.001, χ2=18.662) and friend support (p=0.005, χ2=7.849) among students who attended grammar school and secondary vocational school (S1 Table). Namely, satisfaction with school, schoolwork pressure and friend support were higher in grammar school students, while perceived teacher support was higher in secondary vocational school students. |
| --- | --- | --- | --- | --- |
| Discussion | | | | |
| Key results | 18 | Summarise key results with reference to study objectives | 24 | In this paper, we examined the association between psychosocial school factors and mental health of the first grade secondary school students in Serbia. We found that certain school psychosocial school factors are associated with life satisfaction, depressive symptoms and psychosomatic health complaints, adjusted for demographic, socio- economic and social support variables outside the school environment. |
| Limitations | 19 | Discuss limitations of the study, taking into account sources of potential bias or imprecision. Discuss both direction and magnitude of any potential bias | 27 | Our study had certain limitations: cross-sectional study design that prevents conclusions about the causality between the psychosocial school factors and mental health; not taking into account other factors that could be related to impaired mental health of students, such as objective health status of students and the presence of chronic diseases, or their academic success. |
| Interpretation | 20 | Give a cautious overall interpretation of results considering objectives, limitations, multiplicity of analyses, results from similar studies, and other relevant evidence | 26-27 | The results of this study confirm the complexity of the relationship between psychosocial factors of the school environment with positive and negative aspects of mental health. Overall, most psychosocial factors of the school environment were significantly associated with both positive (life satisfaction) and negative aspects of mental health (symptoms of depression, psychosomatic health complaints). At the same time, the support of classmates and being bullied at school were significant independent predictors for all three observed parameters of mental health. |
| Generalisability | 21 | Discuss the generalisability (external validity) of the study results | 27 | According to our knowledge, this is the first study in Serbia that systematically examines the relationship between psychosocial factors of the school environment and mental health of students on a nationally representative sample. It allows comparison with other countries from the HBSC network using the same methodology and study protocol. |
| Other information | |  | | |
| Funding | 22 | Give the source of funding and the role of the funders for the present study and, if applicable, for the original study on which the present article is based | 29 | The HBSC study in Serbia in 2018, on which this study was based was funded by the Ministry of Health of Serbia. Authors received no funding for conducting this study. |

*Give information separately for cases and controls in case-control studies and, if applicable, for exposed and unexposed groups in cohort and cross-sectional studies.

**Note:** An Explanation and Elaboration article discusses each checklist item and gives methodological background and published examples of transparent reporting. The STROBE checklist is best used in conjunction with this article (freely available on the Web sites of PLoS Medicine at http://www.plosmedicine.org/, Annals of Internal Medicine at http://www.annals.org/, and Epidemiology at http://www.epidem.com/). Information on the STROBE Initiative is available at www.strobe-statement.org.
